# The impacts of baseline ventilator parameters on hospital mortality in acute respiratory distress syndrome treated with venovenous extracorporeal membrane oxygenation: a retrospective cohort study

**DOI:** 10.1186/s12890-017-0520-5

**Published:** 2017-12-08

**Authors:** Meng-Yu Wu, Yu-Sheng Chang, Chung-Chi Huang, Tzu-I Wu, Pyng-Jing Lin

**Affiliations:** 1 Department of Cardiovascular Surgery, Chang Gung Memorial Hospital and Chang Gung University, Taoyuan, Taiwan; 2 School of Traditional Chinese Medicine, Chang Gung University, Taoyuan, Taiwan; 3 Department of Thoracic Medicine, Chang Gung Memorial Hospital and Chang Gung University, Taoyuan, Taiwan; 4 Department of Obstetrics and Gynecology, School of Medicine, College of Medicine, Taipei Medical University, Taipei, Taiwan; 5 Department of Obstetrics and Gynecology, Wan Fang Hospital, Taipei Medical University, Taipei, Taiwan

**Keywords:** Venovenous extracorporeal membrane oxygenation, Adult respiratory distress syndrome, Lung recruitment, Lung-protective mechanical ventilation

## Abstract

**Background:**

Venovenous extracorporeal membrane oxygenation (VV-ECMO) is a valuable life support in acute respiratory distress syndrome (ARDS) in adult patients. However, the success of VV-ECMO is known to be influenced by the baseline settings of mechanical ventilation (MV) before its institution. This study was aimed at identifying the baseline ventilator parameters which were independently associated with hospital mortality in non-trauma patients receiving VV-ECMO for severe ARDS.

**Methods:**

This retrospective study included 106 non-trauma patients (mean age: 53 years) who received VV-ECMO for ARDS in a single medical center from 2007 to 2016. The indication of VV-ECMO was severe hypoxemia (P_a_O_2_/ FiO_2_ ratio < 70 mmHg) under pressure-controlled MV with peak inspiratory pressure (PIP) > 35 cmH_2_O, positive end-expiratory pressure (PEEP) > 5 cmH_2_O, and F_i_O_2_ > 0.8. Important demographic and clinical data before and during VV-ECMO were collected for analysis of hospital mortality.

**Results:**

The causes of ARDS were bacterial pneumonia (*n* = 41), viral pneumonia (*n* = 24), aspiration pneumonitis (*n* = 3), and others (*n* = 38). The median duration of MV before ECMO institution was 3 days and the overall hospital mortality was 53% (*n* = 56). The medians of PaO_2_/ FiO_2_ ratio, PIP, PEEP, and dynamic pulmonary compliance (PC_dyn_) at the beginning of MV were 84 mmHg, 32 cmH_2_O, 10 cmH_2_O, and 21 mL/cmH_2_O, respectively. However, before the beginning of VV-ECMO, the medians of PaO_2_/ FiO_2_ ratio, PIP, PEEP, and PC_dyn_ became 69 mmHg, 36 cmH_2_O, 14 cmH_2_O, and 19 mL/cmH_2_O, respectively. The escalation of PIP and the declines in PaO_2_/ FiO_2_ ratio and PC_dyn_ were significantly correlated with the duration of MV before ECMO institution. Finally, the duration of MV (OR: 1.184, 95% CI: 1.079–1.565, *p* < 0.001) was found to be the only baseline ventilator parameter that independently affected the hospital mortality in these ECMO-treated patients.

**Conclusion:**

Since the duration of MV before ECMO institution was strongly correlated to the outcome of adult respiratory ECMO, medical centers are suggested to find a suitable prognosticating tool to determine the starting point of respiratory ECMO among their candidates with different duration of MV.

**Trial registration:**

This study reported a health care intervention on human participants and was retrospectively registered. The Chang Gung Medical Foundation Institutional Review Board approved the study (no. 201601483B0) on November 23, 2016. All of the data were extracted from December 1, 2016, to January 31, 2017.

## Background

Extracorporeal Membrane Oxygenation (ECMO) is currently an important life support for acute respiratory distress syndrome (ARDS) in adult patients [1, 2]. According to the 2016 international report of the Extracorporeal Life Support Organization (ELSO) Registry, 58% of the adult patients receiving ECMO for severe respiratory failure can be saved and discharged from hospital [3]. This report also reveals that about 90% of the 9812 ECMO runs for adult respiratory failure are in venovenous (VV)-associated configurations [3]. The niche of VV-ECMO in the management of ARDS is to provide a pre-pulmonary blood gas exchange to the venous blood and reduce the patient’s dependence on pulmonary ventilation [2].While the patient’s dependence on pulmonary ventilation is reduced, the risk and the severity of ventilator-induced lung injury (VILI) can theoretically be mitigated. Although the popularity of adult respiratory ECMO is continuously increasing, the applications of ECMO are still limited in large medical centers and reserved for the most advanced diseases [1]. The discrepancy in user experience leads to considerable controversies about the timing of respiratory ECMO among experts worldwide. Currently, the timing of respiratory ECMO is mostly determined by the severity of hypoxemia which is represented by the ratio of arterial oxygen tension (PaO_2_) to the fraction of inspired oxygen (FiO_2_) under mechanical ventilation (MV). In the ELSO Guidelines for Adult Respiratory Failure, the suggested threshold value of PaO_2_/FiO_2_ (PF) ratio for ECMO institution is 100 mmHg or less [4]. However, under the inclusion criteria based on PF ratio, patients with a relatively slow-progressive disease may experience a significant escalation in the driving force of MV before their PF ratio can finally meet the threshold value for ECMO [5]. Since the therapeutic goal of respiratory ECMO is to reduce the negative influence of MV on the success of adult respiratory ECMO, the starting point of respiratory ECMO should also take the determinant ventilator parameters into consideration. Therefore, the study was aimed at identifying the baseline ventilator parameters which were independently associated with hospital mortality in patients receiving VV-ECMO for severe ARDS.

## Methods

### Settings and patients

From March 2007 to March 2016, a total of 151 adult patients received VV-ECMO for advanced respiratory support at Chang Gung Memorial Hospital Linko Branch. The university-affiliated hospital is a tertiary referral center with 3400 beds. To avoid the influences of trauma or surgery on blood coagulation and compliance of the respiratory system, we only enrolled 106 adult non-trauma patients who had a single run of VV-ECMO and survived on VV-ECMO >24 h in this retrospective study. This study was conducted in accordance with the amended Declaration of Helsinki. The ethics committee of the Chang Gung Medical Foundation approved this protocol (CGMF IRB no. 201601483B0) and waived the necessity of individual patient consent.

### Managements of adult VV-ECMO

Our techniques of applying MV and ECMO to improve hypoxemia in patients with ARDS were described previously [5–9]. Before ECMO is considered, patients with ARDS were treated with the lung-protective MV and paralyzed with neuromuscular blockers. Our lung-protective MV is pressure-controlled ventilation which uses a peak airway pressure (PIP) less than 35 mmHg to drive a tidal volume (V_T_) no more than 6 ml/ kg. To prevent carbon dioxide (CO_2_) retention and oxygen toxicity, the respiratory rate, the positive end-expiratory pressure (PEEP), and the F_i_O_2_ of MV are set at 20 to 25/min, 10 to 18 cmH_2_O, and less than 0.8, respectively. VV-ECMO would be delivered to suitable candidates if they required a higher PIP and FiO_2_ for maintaining a PF not less than 70 mmHg. Nevertheless, VV-ECMO was contraindicated in candidates showing (1) uncontrolled hemorrhages, (2) major brain damages, and (3) significant hemodynamic instability. The definition of significant hemodynamic instability here was circulatory shock (systolic arterial blood pressure < 90 mmHg) that required a high-dosed inotrope/vasopressor therapy (dopamine >15 mcg/kg/min, epinephrine >0.1 mcg/kg/min, or norepinephrine >0.1 mcg/kg/min). Our ECMO devices include a centrifugal pump [Capiox emergent bypass system (Terumo, Tokyo, Japan) or Bio-console 560 system (Medtronic, Minneapolis, MN, USA)], an oxygenator with silicone membrane (Medtronic, Minneapolis, MN, USA) or polymethylpentene membrane (Terumo Capiox-SX or Medos Hilite 7000), and two vascular cannulae (DLP Medtronic, Minneapolis, MN, USA). We conduct VV-ECMO via percutaneous cannulation of the common femoral vein (inflow, with a cannula of 19–23 French) and the right internal jugular vein (outflow, with a cannula of 17–21 French). After implantation of VV-ECMO, we initially maximize the sweep gas flow (10 L/min, pure oxygen) to rapidly remove CO_2_, and gradually increase the ECMO pump flow to achieve a steady flow that carries the best pulse oximetry-detected oxyhemoglobin saturation (SpO_2_). To rest the injured lungs, we gradually downgrade the original MV settings to a non-injurious level (PIP ≤ 30 cmH_2_O and MV FiO_2_ ≤ 0.4). According to the data of arterial and post-oxygenator blood gas samplings, we dynamically adjust the flows of ECMO to provide an optimal SpO_2_ (> 90%) and arterial oxyhemoglobin saturation (SaO_2_; > 85%). A modest anticoagulation (activated partial thromboplastin time between 40 and 55 s) is achieved with systemic heparinization except in hemorrhagic patients. We would try to wean the improved patients from VV-ECMO as long as the arterial oxygenation could be maintained with small V_T_ and ventilator FiO_2_ no more than 0.6.

### Data collection

We retrospectively reviewed the electronic medical records of every patient and collected their relevant demographic and clinical data before and during ECMO run. Since sequential organ failure assessment (SOFA) score [10] and respiratory extracorporeal membrane oxygenation survival prediction (RESP) score [11] have become our major prognosticating tools for adult respiratory ECMO now, we collected the essential data to calculate the two scores in each patient. Therefore the following variables were collected: age, gender, body weight and height, acute respiratory diagnosis (viral pneumonia, bacterial pneumonia, asthma, trauma/burn, aspiration, and others), immunocompromised status (malignancy, organ transplantation, liver cirrhosis Child B or C, or autoimmune diseases requiring long-term immunosuppressive therapy), non-pulmonary infection, duration of MV before institution of VV-ECMO, MV settings [measured ventilation volume, PIP, mean airway pressure (MAP), PEEP, dynamic driving pressure, dynamic pulmonary compliance (PC_dyn_), and oxygen index (OI)] soon (1 to 2 h) after institution of MV and just (1–2 h) before institution of VV-ECMO, special medications (neuromuscular blockers, bicarbonate or vasopressors) before institution of VV-ECMO, the latest results of blood tests (arterial blood gas sampling, blood cell counts, creatinine, and total bilirubin) before institution of VV-ECMO, durations of hospital and ECMO stay, and outcomes (survived or died in hospital). In the survivors, we also collected the MV settings just before and after 24 h of ECMO removal. The V_T_ was defined as the measured ventilation volume dividing by the ideal body weight. The dynamic driving pressure was defined as the difference of PIP and PEEP. PC_dyn_ was defined as the measured ventilation volume dividing by the difference of PIP and PEEP. The OI was defined as the product of MAP and FiO_2_ dividing by PaO_2_. The baseline value of a given variable was the value obtained just before institution of VV-ECMO. For our practical purposes, we made some modifications of the definitions in the original RESP score. First, we assigned the patients with fungal pneumonia to the category of bacterial pneumonia, because fungal pneumonia was not a category of diagnosis in RESP score and the number of fungal pneumonia in our patient cohort was small. Second, we excluded the item of nitric oxide inhalation because this information was often missing in our patient cohort. Third, we assigned a SOFA neurological assessment score to each patient according to his/her neurological status before sedation [12].

### Outcome measures

The endpoint of this study was to identify the predictors of hospital mortality in adult respiratory ECMO among the baseline ventilator parameters.

### Statistical analyses

Statistical analyses were performed with SPSS for Windows (Version 21, IBM, Inc., NY, USA). For all analyses, the statistical significance was set at *p* < 0.05. The independent T-test or the Mann-Whitney *U* test was used for univariate comparison of numerical variables. The Chi-square or Fisher’s exact test was used for univariate comparison of categorical variables. The data were presented as mean (± standard deviation) for numerical variables with normal distribution or median (interquartile range; IQR) for numerical variables without normal distribution. The categorical data were presented as number (percentage). The multivariate logistic regression method was used to identify the independent predictors of hospital mortality and to build up the mortality risk prediction model. All variables with a *p* < 0.05 in univariate tests were firstly processed by the logistic regression method with a backward stepwise selection procedure. The variables showed a *p* < 0.05 in the logistic regression process were re-tested by the logistic regression method with a forward stepwise selection procedure to build the final prediction model. The final model was evaluated by the Hosmer-Lemeshow test and the receiver operating characteristic curve analysis for its goodness-of-fit and the predictive power for hospital mortality.

## Results

### Patient characteristics

**Fig. 1:**
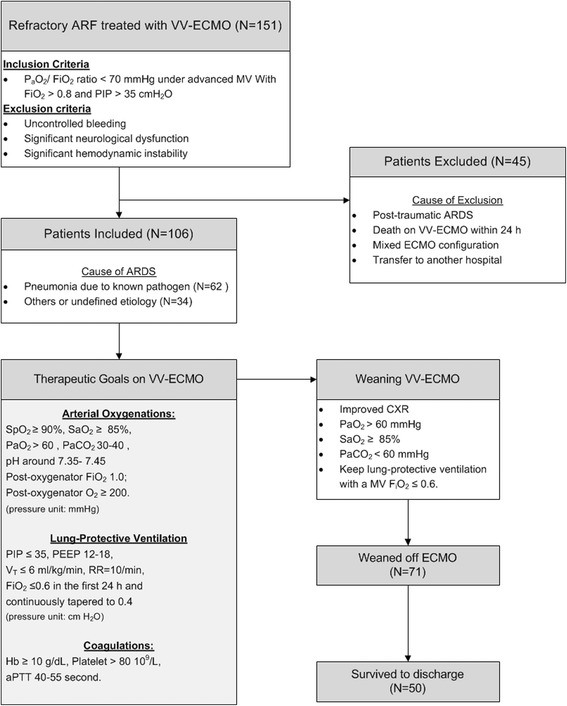
Flow chart of patient distribution and managements during venovenous extracorporeal membrane oxygenation. ARF: Acute respiratory failure. FiO2: The fraction of inspired oxygen. PaO2: Arterial oxygen tension. PEEP: Positive end-expiratory pressure. PIP: Peak inspiratory pressure. RR: Respiratory rate. SaO2: Arterial oxygen saturation; SpO2: Oxyhemoglobin saturation by pulse oximetry. V_T_: Tidal volume. VV-ECMO: Venovenous extracorporeal membrane oxygenation

Our therapeutic protocol and associated patient distribution are presented in fig. 1. The results of univariate comparisons of important demographic and clinical data between the survivors and the non-survivors are presented in Table 1. The causes of ARDS were categorized into 4 groups: bacterial pneumonia (*n* = 41; three were fungal pneumonia, and the top three found bacteria were *Staphylococcus aureus, Pseudomonas aeruginosa,* and *Acinetobacter baumannii*), viral pneumonia (*n* = 24; all influenza type A), aspiration pneumonitis (*n* = 3), and others (*n* = 38). The “others” group included (1) pneumonia without identifiable pathogens (*n* = 24); (2) pulmonary hemorrhage caused by autoimmune vasculitis (*n* = 2); (3) pneumonia after near-drowning (*n* = 1); and (4) pulmonary edema due to acute on chronic renal failure (*n* = 4), acute pancreatitis (*n* = 3), or after percutaneous interventions (*n* = 4; 3 for cardiac lesions and 1 for cerebral aneurysm). All patients received VV-ECMO in our institution. Three patients received MV support before they were transferred to our hospital, and the duration of MV support before their ER admission were 10 h, 18 h, and 4 days. Diagnoses in the 37 immunocompromised patients were malignancies (*n* = 16; 15 solid tumor and 1 lymphoma), autoimmune diseases (*n* = 10; 2 dermatomyositis, 2 idiopathic thrombocytopenia, 2 granulomatosis with polyangiitis, 1 psoriatic arthritis, 1 rheumatoid arthritis, 1 systemic lupus erythematosus, and 1 Graves’disease), immunosuppressive therapy in solid organ transplantation (*n* = 8; 6 liver transplantation and 2 renal transplantation), advanced liver cirrhosis (*n* = 2), and steroid therapy in asthma (*n* = 1). Fifty-six patients died in hospital and 35 of them died on VV-ECMO. Six patients died on VV-ECMO due to hemorrhagic complications including intracranial hemorrhages (n = 2), intra-abdominal or retroperitoneal hemorrhages (*n* = 1), and gastrointestinal tract hemorrhages (*n* = 4). The multiple-organ failure syndrome with sepsis was the cause of death for the other non-survivors. The results of univariate comparisons of ventilator parameters between the survivors and non-survivors are also presented in Table 2. These parameters were obtained soon after the beginning of MV and just before the beginning of VV-ECMO. The 3 patients receiving MV support in other hospital were excluded from the analysis of ventilator parameters just after the beginning of MV. Differences between the pre-ECMO and the early MV data of a given ventilator parameter were also calculated to present the deterioration of pulmonary function and the corresponding escalation of ventilation pressures before ECMO institution.

**Table 1:** Patient characteristics before venovenous extracorporeal membrane oxygenation

	All (*n* = 106)	Survivor (*n* = 50)	Non-survivor (*n* = 56)	*P*
Age (year)	53 ± 15	51 ± 15	55 ± 15	0.11
Male	71 (67)	35 (70)	36 (64)	0.53
Predicted body weight^a^ (kg)	55 ± 14	55 ± 16	55 ± 11	0.89
Hospital day before ECMO	6 (1–14)	2(1–9)	11(4–20)	<0.001*
Mechanical ventilation before ECMO (day)	3 (1–9)	1(0–4)	6(1–12)	<0.001*
Cause of ARDS				
Viral pneumonia	24 (21)	11 (22)	13 (23)	0.88
Bacterial pneumonia	38 (37)	16 (32)	22 (39)	0.54
*Fungal pneumonia*	3 (3)	0	3 (6)	0.25
Aspiration pneumonitis	3 (3)	3 (6)	0	0.10
Other acute respiratory diagnoses	38 (36)	20 (40)	18 (32)	0.40
Acute associated infection	24 (21)	11 (22)	13 (23)	0.88
Immunocompromised status^b^	37 (35)	11 (22)	26 (46)	0.008*
Renal failure requiring dialysis	28 (26)	13 (26)	15 (27)	0.93
Creatinine (mg/dL)	1.6 (0.8–3.4)	2.8 ± 3.3	2.3 ± 2.3	0.36
Total bilirubin (mg/dL)	1.5 ± 2.5	1.1 ± b1.2	1.9 ± 3.2	0.14
Platelet count (10^9^/L)	137 (83–218)	172 (113–237)	106 (60–161)	0.002*
Hemoglobin (g)	10 (9–12)	11 (9–12)	10 (9–11)	0.08
SOFA score	10 (8–11)	9 (7–10)	10 (9–12)	0.002*
RESP score	2 ± 3	2 ± 3	1 ± 3	0.05*

Data were presented as mean ± standard deviation in normal-distributed numerical variables, median (interquartile range) in numerical variables not normal-distributed, and *n* (%) in categorical variables. *ECMO* Extracorporeal membrane oxygenation, *SOFA* sequential organ failure assessment, *RESP score* Respiratory extracorporeal membrane oxygenation survival prediction score, *ARDS* Acute respiratory distress syndrome. ^a^ Ideal body weight is calculated by the ARDSnet formulas. ^b^Immunocompromised status includes hematologic malignancy, solid tumor, solid organ transplantation, liver cirrhosis Child B or C, or autoimmune diseases requiring long-term immunosuppressive therapy

*p < 0.05. (in the comparisons between survivors and non-survivors)

**Table 2:** Ventilator parameters before venovenous extracorporeal membrane oxygenation

	All (*n* = 106)	Survivor (*n* = 50)	Non-survivor (*n* = 56)	*P*
Just after intubation				
P_a_O_2_/F_i_O_2_ (mmHg)	112 ± 76	90 ± 55	129 ± 85	0.009*
Peak inspiratory pressure (cmH_2_O)	33 ± 6	32 ± 5	33 ± 6	0.86
Mean airway pressure (cmH_2_O)	18 ± 4	18 ± 4	18 ± 5	0.85
PEEP (cmH_2_O)	12 ± 3	12 ± 3	12 ± 3	0.56
Dynamic driving pressurea^a^ (cmH_2_O)	21 ± 5	21 ± 5	22 ± 5	0.86
Measured tidal volume (ml/kg)	7.7 ± 2.3	7.7 ± 2.3	7.8 ± 2.3	0.73
Dynamic compliance^b^ (ml/cmH_2_O)	22 ± 9	23 ± 10	21 ± 8	0.15
Oxygen index^c^	25 ± 19	29 ± 22	21 ± 15	0.11
Just before ECMO				
P_a_O_2_/F_i_O_2_ (mmHg)	72 ± 17	72 ± 19	72 ± 16	0.93
Peak inspiratory pressure (cmH_2_O)	36 ± 6	35 ± 5	37 ± 6	0.16
Mean airway pressure (cmH_2_O)	21 ± 4	21 ± 4	22 ± 4	0.13
PEEP (cmH_2_O)	14 ± 3	14 ± 3	14 ± 3	0.79
Dynamic driving pressure^a^ (cmH_2_O)	22 ± 6	21 ± 5	23 ± 6	0.22
Measured tidal volume (ml/kg)	6.7 (6–7.8)	6.8 (6.1–8.7)	6.7 (5.8–7.7)	0.22
Dynamic compliance^b^ (ml/cmH_2_O)	19 (15–23)	21 (15–25)	17 (12–21)	0.01*
Oxygen index^c^	39 ± 13	39 ± 14	38 ± 13	0.66
Difference				
∆ P_a_O_2_/F_i_O_2_ (mmHg)	−16 (−71–9)	0 (−51–21)	−31 (−95–5)	0.009*
∆ Peak inspiratory pressure (cmH_2_O)	4 ± 6	4 ± 6	4 ± 7	0.18
∆ PEEP (cmH_2_O)	3 ± 4	3 ± 3	3 ± 4	0.92
∆ Dynamic driving pressure^a^ (cmH_2_O)	1 ± 6	1 ± 6	1 ± 7	0.33
∆ Measured tidal volume (ml/kg)	−0.3 (−1.9–0.8)	0 (−1.5–1.2)	−0.8 (−1.9–0.2)	0.05*
∆ Dynamic compliance^b^ (ml/cmH_2_O)	−2 ± 11	−1 ± 11	−3 ± 11	0.72
∆ Oxygen index^c^	14 ± 2	10 ± 21	17 ± 19	0.35

Data were presented as mean ± standard deviation in normal-distributed numerical variables, median (interquartile range) in numerical variables not normal-distributed, and n (%) in categorical variables. *ECMO* Extracorporeal membrane oxygenation, *PEEP* Positive end expiratory pressure. ^a^Driving pressure = (Peak inspiratory pressure – Positive end-expiratory pressure)

^b^Dynamic pulmonary compliance = Measured tidal volume/ Driving pressure

^c^Oxygen index = [(Mean airway pressure□x□FiO_2_□x 100)/ arterial oxygen tension]

*p < 0.05. ∆: The data obtained before ECMO - the data obtained after intubation

### Multivariate prediction model of hospital death

**Fig. 2:**
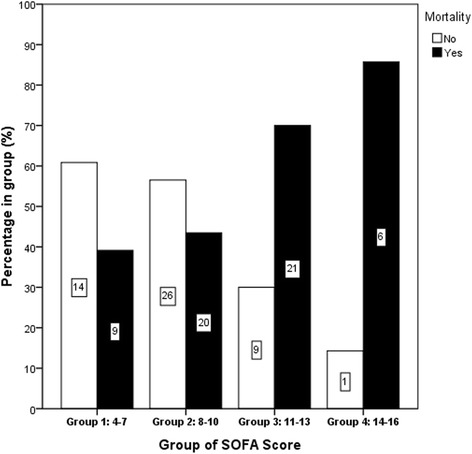
Observed mortality rates among patients categorized by the baseline sequential organ failure assessment (SOFA) score. The case number in each group is also presented

According to the results of multivariate analysis, the pre-ECMO duration of MV [Odd ratio (OR): 1.184; 95% confident interval (CI): 1.079–1.565, *p* < 0.001] and the pre-ECMO SOFA score (OR: 1.299; 95% CI: 1.077–1.302; *p* = 0.006) were identified to be the independent predictors of hospital mortality in adult non-trauma patients who received VV-ECMO for severe ARDS. The mortality prediction model built with these factors was presented as follows: Predicted mortality (y) = ℮^X^ / (1 + ℮^X^). X = −3.218 + 0.169 × (days of MV before institution of VV-ECMO) + 0.262 × (SOFA score before institution of VV-ECMO). The model explained 30% (Nagelkerke R^2^) of the variance in hospital mortality and correctly classified 68.9% of the cases (sensitivity: 66.1%; specificity: 72%). This predictive model also fitted the dataset well (Hosmer-Lemeshow test: χ ^2^ = 7.526, *p* = 0.376) and showed a fair predictive power of hospital mortality (c-index: 0.763, *p* < 0.001, 95% CI: 0.674–0.851). Figure 2 demonstrates the observed hospital mortality rates among patients grouped by their baseline SOFA score.

### Serial changes of arterial oxygenation during the course of treatment

**Fig. 3:**
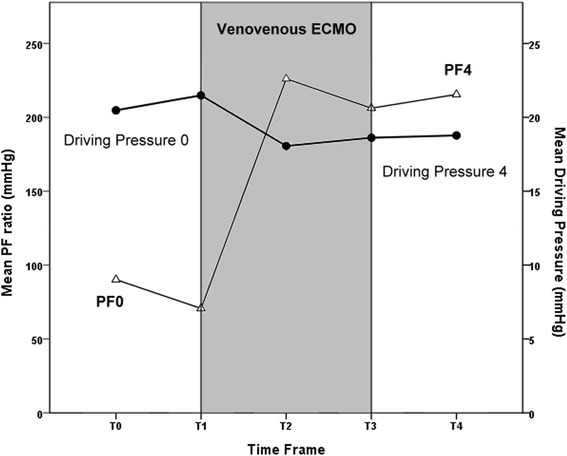
Survivors’ trends of PF ratio and dynamic driving pressure during the support of VV-ECMO. (T_0_: 1–2 h after intubation for MV, T_1_: 1–2 h before ECMO cannulation, T_2_:24 h after ECMO institution, T_3_: 1–2 h before ECMO decannulation, and T_4_: 24 h after ECMO removal)

To get a deeper understanding of the influences of VV-ECMO combining lung-protective MV on arterial oxygenation during the course of treatment, we collected the ventilator parameters at several time points (T0: 1 to 2 h after intubation for MV, T1: 1 to 2 h before ECMO cannulation, T2:24 h after ECMO institution, T3: 1 to 2 h before ECMO decannulation, and T4: 24 h after ECMO removal) for analysis and the results are demonstrated in Table 3. It is notable that only the survivors could go through the whole treatment and show data in all of the 5 points. The survivors’ trends of PF ratio and dynamic driving pressure are also illustrated in fig. 3.

**Table 3:** The serial changes of pressure settings of mechanical ventilation and index of arterial oxygenation during and after removal of venovenous extracorporeal membrane oxygenation

	24 h after ECMO institution (T2)	Just before ECMO removal. (T3)	24 h after ECMO removal (T4)
Survivors (*n* = 50)			
P_a_O_2_/F_i_O_2_ (mmHg)	226 ± 85*	206 ± 89	216 ± 94
Peak inspiratory pressure (cmH_2_O)	30 ± 5	30 ± 5	24 ± 13
Mean airway pressure (cmH_2_O)	17 ± 3	17 ± 4	17 ± 4
PEEP (cmH2O)	12 (10–14)	10 (10–12)	12 (10–12)
Dynamic driving pressure^a^ (cmH2O)	18 ± 6	19 ± 5	19 ± 6
Measured tidal volume (ml/kg)	7 ± 1	8 ± 2	7 ± 4
Dynamic compliance^b^ (ml/cmH_2_O)	24 ± 9*	28 ± 12	22 ± 15
Oxygen index^c^	8 ± 4*	10 ± 6	10 ± 6
ECMO outflow PaO_2_ (mmHg)	391 (338–449)	50 (38–133)	–
ECMO outflow PaCO_2_ (mmHg)	35 (28–40)	40 (36–59)	–
ECMO outflow O_2_ saturation (%)	100	100	–
Non-Survivors (n = 56)			–
P_a_O_2_/F_i_O_2_ (mmHg)	164 ± 84*	–	–
Peak inspiratory pressure (cmH_2_O)	32 ± 5	–	–
Mean airway pressure (cmH_2_O)	18 ± 4	–	–
PEEP (cmH2O)	13 ± 3	–	–
Dynamic driving pressure^a^ (cmH2O)	19 ± 6	–	–
Measured tidal volume (ml/kg)	6 ± 3	–	–
Dynamic compliance^b^ (ml/cmH_2_O)	19 ± 10*	–	–
Oxygen index^c^	14 ± 7*	–	–
ECMO outflow PaO_2_ (mmHg)	408 ± 79	–	–
ECMO outflow PaCO_2_ (mmHg)	36 ± 6	–	–
ECMO outflow O_2_ saturation (%)	100	–	–

Only the survivors showed data recorded just before and 24 h after ECMO removal

Data were presented as mean ± standard deviation in normal-distributed numerical variables, median (interquartile range) in numerical variables not normal-distributed, and n (%) in categorical variables. *ECMO* Extracorporeal membrane oxygenation, *PEEP* Positive end expiratory pressure

*The mean values of a specific variable in the T2 column are significant different between the survivors and non-survivors while analyzed by independent T-test (*p* < 0.05)

^a^Driving pressure = (Peak inspiratory pressure – Positive end-expiratory pressure)

^b^Dynamic pulmonary compliance = Measured tidal volume/ Driving pressure

^c^Oxygen index = [(Mean airway pressure□x□FiO_2_□× 100)/ arterial oxygen tension]

## Discussion

This study revealed that the duration of MV before ECMO institution was the only baseline ventilator parameter which was independently associated with hospital mortality in non-trauma patients receiving VV-ECMO for severe ARDS, although the mechanism of how a prolonged duration of MV could jeopardize the survival in these ECMO-treated patients was still unclear. In fact, this duration is a reciprocal measure of the declining rate of PF ratio from the value obtained at the beginning of MV to the given threshold value for ECMO. Clinically, this declining rate of PF ratio is significantly affected by the etiology of ARDS, patient characteristics, and the institutional experience on advanced modes of MV, as the conclusion of CESAR trial [11, 13]. These uncertainties make the suitable duration of MV before ECMO individualized among institutions. Nevertheless, limitation of this duration should still be important to adult candidates of respiratory ECMO. According to Table 2, regardless of the value of initial PF ratio, most of our patients were found to have severely non-compliant lungs at the beginning of MV. The mean PC_dyn_ measured at the beginning of MV was 22 cm H_2_O in all of the patients, which was only accounted for 10% or less of the normal value [14]. This finding implied that these patients were very sensitive and vulnerable to the cyclic pulmonary manipulation of MV [15–17]. When the disease progresses and involves more pulmonary segments, clinicians often need to open the collapsed segments with an increased driving pressure of MV to maintain an acceptable blood-gas exchange. This attempt of lung recruitment may considerably increase the amount of dead space ventilation rather than effectively improve the blood-gas exchange, since the local perfusion of the distended segments may drop, as demonstrated by Gattinoni et al. [18]. The over-distended lungs may also increase the intra-thoracic pressure and compromise the cardiac output [19]. Therefore, if available and not contraindicated, VV-ECMO combining lung-protective MV is a valuable strategy to reduce pulmonary manipulation and reverse some of the pulmonary segments performing dead space ventilation under high-pressure MV to the segments performing effective blood-gas exchange under a reduced inspiratory pressure. However, the benefit of VV-ECMO is often small in patients with prolonged ventilation (often >7 days) or severe multiple organ dysfunctions [2]. From this viewpoint, it should be important for ECMO centers to have a practical tool to determine the starting point of respiratory ECMO among candidates with different duration of MV. Technically, there are three common ways to reduce the risk of a prolonged MV before ECMO institution. The first is choosing an arbitrary deadline which is set according to general experiences, such as a 7-day period [20]. The second is loosening the threshold value of PF ratio for ECMO from 100 mmHg to 150 mmHg, as per the suggestion of ELSO Guidelines. The third is creating a risk assessment model for a multi-axial evaluation, as is our choice here.

What interested us was that the baseline PIP could not be identified as a prognostic predictor of adult respiratory ECMO in the current study, which was different from the suggestion of RESP score. The RESP score is derived from a retrospective analysis of 2355 adult patients in ELSO’s registry and reveals that the baseline PIP, with a threshold value of 42 cmH_2_O, is a predictive factor for hospital mortality in adult patients receiving respiratory ECMO [11]. However, in the current study including 106 patients, the patients with a baseline PIP > 42 cm H_2_O (*n* = 14) showed a significantly lower hospital mortality rate than the patients with a baseline PIP ≤ 42 cm H_2_O (21% vs. 58%, *p* = 0.02). We thought that the discrepancy in sample size between the two studies should have some connection to this unexplained result. In the original study of RESP score [11], the odds ratio of baseline PIP for hospital survival is close to 1.0 (0.992). Furthermore, the medians of baseline PIP were surprisingly both the same (36 cmH_2_O) in the survivors (*n* = 1338) and non-survivors (*n* = 1017). Therefore, the baseline PIP might not be a very suitable criterion to initiate respiratory ECMO. Some researchers suggest that the baseline plateau pressure (P_plat_) is also a valuable indicator used for this purpose [21]. Although P_plat_ is a better airway pressure than PIP for measuring the pressure applied to lung itself during MV, we were unable to reproduce the above-mentioned result because the data of P_plat_ were severely incomplete in this retrospective study due to unknown reason.

Although the impact of the baseline pressure settings of MV remains equivocal on the outcomes of adult respiratory ECMO, an unreduced static or dynamic driving pressure of MV during the first three days of adult respiratory ECMO is recently reported to be a predictor of hospital mortality in these ECMO-treated patients [22, 23]. When the results of these updated investigations on adult respiratory ECMO are reviewed together, researchers may find that there seem to be some links among the duration of MV before ECMO institution, the driving pressure of MV during ECMO, and the outcomes of ECMO. According to Tables 2 and 3, all of the patients showed an improvement in arterial oxygenation and a downgrade of driving pressure of MV during the first 24 h of ECMO institution. Since the collapsed alveoli should be difficult to be re-opened by a decreased driving pressure, we could assume that the improvement in arterial oxygenation should depend on the oxygenator and the non-collapsed alveoli. However, as shown in fig. 3, only patients maintaining the trend of improvement in arterial oxygenation throughout the ECMO support could eventually wean ECMO and survive. It is notably that the pulmonary compliance remained impaired in the survivors after a successful rescue of ECMO. These findings implied that the size of the non-collapsed pulmonary segments before ECMO should be one of the key contributors to the patient’s progression on ECMO and the outcome. To keep the non-collapsed pulmonary segments opened under a reduced driving pressure during ECMO, the decrease in the driving pressure during the early support of ECMO was achieved by a reduction in PIP rather than in PEEP. Actually, choosing a moderate high PEEP is also an important issue in patients treated with lung-protective MV [17]. Despite not used in this study, an esophageal balloon is considered to be a useful tool to measure the serial changes of transpulmonary pressure and suggested an appropriate level of PEEP and driving pressure in this scenario [24]. In general, ventilating the patients with an unreduced driving pressure is not an effective solution to improve hypoxemia on VV-ECMO, since this behavior means that the clinicians still want to recruit the collapsed segments. In cases with a refractory hypoxemia during VV-ECMO, the circuit of ECMO should be rearranged to a hybrid configuration which may promptly correct the arterial hypoxemia and allow a decrease in driving pressure [25]. However, significant hypoxemia may recur during the weaning process of ECMO in any configuration if the pulmonary parenchyma is extensively damaged and the residual reservoir is below the requirement for surviving without ECMO.

The major limitations of this study are its retrospective design and moderate sample size. This study did not provide a comprehensive discussion of adult respiratory ECMO since only non-trauma patients treated with pressure-controlled ventilation and VV-ECMO in a single center were included. Further collaborative and prospective studies that adopt new techniques to obtain novel respiratory parameters in a large cohort of ECMO-treated ARDS patients are necessary to validate our hypothesis and determine the influences of baseline ventilator parameters on outcomes of adult respiratory ECMO.

## Conclusion

Among the baseline ventilator parameters included in this study, duration of MV was the only parameter independently associated with hospital mortality in adult non-trauma patients treated with VV-ECMO. The benefit of VV-ECMO was small in patients with prolonged ventilation or severe multiple organ dysfunctions. Therefore, medical centers were suggested to find a suitable prognosticating tool to determine the starting point of respiratory ECMO among their candidates with different duration of MV.

## Abbreviations

AaDO2: Alveolar-arterial oxygen difference
APTT: Activated partial thromboplastin time
ARDS: Acute respiratory failure
ECMO: Extracorporeal membrane oxygenation
FiO_2_: Fraction of inspiratory oxygen
LPV: Lung-protective ventilation
MV: Mechanical ventilation
OI: Oxygen Index
PaCO_2_: Arterial carbon dioxide tension
PaO_2_: Arterial oxygen tension
PEEP: Positive end-expiratory pressure
PIP: Peak inspiratory pressure
PT: Prothrombin time
RV: Right ventricle
SaO2: Arterial oxygen saturation
SOFA: Sequential organ failure assessment
SpO2: Oxyhemoglobin saturation by pulse oximetry
VA: Venoarterial
VILI: Ventilator induced lung injury
VV: Venovenous
